# Association between serum uric acid and bone mineral density in males from NHANES 2011–2020

**DOI:** 10.1038/s41598-024-52147-8

**Published:** 2024-02-21

**Authors:** Renwei Wang, Yao Gao, Peng Wang, Chunru He, Hao Lu

**Affiliations:** 1 Department of Orthopedic, Linfen Central Hospital, Linfen, 041000 China; 2 Department of Orthopedic, Lin Fen Central Hospital Affiliated to ChangZhi Medical College, Linfen, China; 3 Department of Spinal Surgery, The First Affiliated Hospital of Guangzhou University of Chinese Medicine, Guangzhou, 510405 China

**Keywords:** Biomarkers, Endocrinology

## Abstract

Currently, the relationship between serum uric acid (SUA) and bone mineral density (BMD) in men remains controversial. This study aims to investigate the relationship between SUA and lumbar spine BMD in American men using data from the National Health and Nutrition Examination Survey (NHANES). A total of 6254 male subjects aged 12–80 years (mean age 35.52 ± 14.84 years) in the NHANES from 2011 to 2020 were analyzed. SUA was measured by DxC using the timed endpoint method, and lumbar spine BMD was measured by dual-energy X-ray absorptiometry (DXA). Multivariate linear regression models were used to explore the relationship between SUA and BMD by adjusting for age, race/Hispanic origin, drinking behavior, smoking behavior, physical activity, body mass index (BMI), poverty-to-income ratio (PIR), total protein, serum calcium, cholesterol, serum phosphorus, and blood urea nitrogen. After correcting for the above confounders, it was found that SUA was positively associated with lumbar spine BMD in the range of SUA < 5 mg/dL (β = 0.006 95% CI 0.003–0.009, P < 0.001), and BMD of individuals in the highest quartile of SUA was 0.020 g/cm^2^ higher than those in the lowest quartile of SUA (β = 0.020 95% CI 0.008–0.032, P = 0.003). This study showed that SUA was positively correlated with lumbar spine BMD in American men within a certain range. This gives clinicians some insight into how to monitor SUA levels to predict BMD levels during adolescence when bone is urgently needed for growth and development and during old age when bone loss is rapid.

## Introduction

Osteoporosis, a systemic skeletal disease characterized by low bone mass and microarchitectural deterioration of bone tissue, with a consequent increase in bone fragility and susceptibility to fractures^1^, is common and increases as the population ages^2^. It causes a large number of bone-related diseases and increases mortality and health care costs^3^. Moreover, lumbar fractures are the most typical osteoporotic fractures, and they are strongly associated with lower bone mineral density (BMD) in the lumbar spine^4^. Therefore, it is particularly valuable to investigate the factors associated with reduced BMD due to the increasing economic and social burden caused by osteoporosis.

Uric acid is the end product of purine metabolism^5^, and serum uric acid (SUA) disorders are major risk factors for gout^6^. Numerous studies have concluded that hyperuricemia is also a predictor of the development of hypertension, metabolic syndrome, type 2 diabetes, cardiovascular disease, and renal disease^5,7–9^. However, an increasing amount of experimental and clinical evidence suggests that uric acid, as an antioxidant in humans, has an important role. A review provides current evidence on the antioxidant role of uric acid and suggests its potential therapeutic effects as a marker of oxidative stress and as an antioxidant^10^. Due to its antioxidant properties, SUA may enhance bone density by inhibiting osteoclast bone resorption and promoting osteoblast differentiation^11^. A meta-analysis of data on BMD, osteoporosis, and fractures in people with high and low SUA concentrations confirmed that there was a correlation between hyperuricemia and BMD, and uric acid played a protective role in disorders of bone metabolism^12^.

In conclusion, there is limited evidence on the relationship between SUA and BMD in men. Exploring the above relationship in different age groups and races can provide important information for individuals, clinicians, and health care providers to develop osteoporosis prevention and treatment strategies. Therefore, SUA and gout were selected as indicators, and a representative accredited sample from the National Health and Nutrition Examination Survey (NHANES) was used to assess the relationship between SUA and BMD in American men.

## Materials and methods

### Study design and population

**Figure 1:**
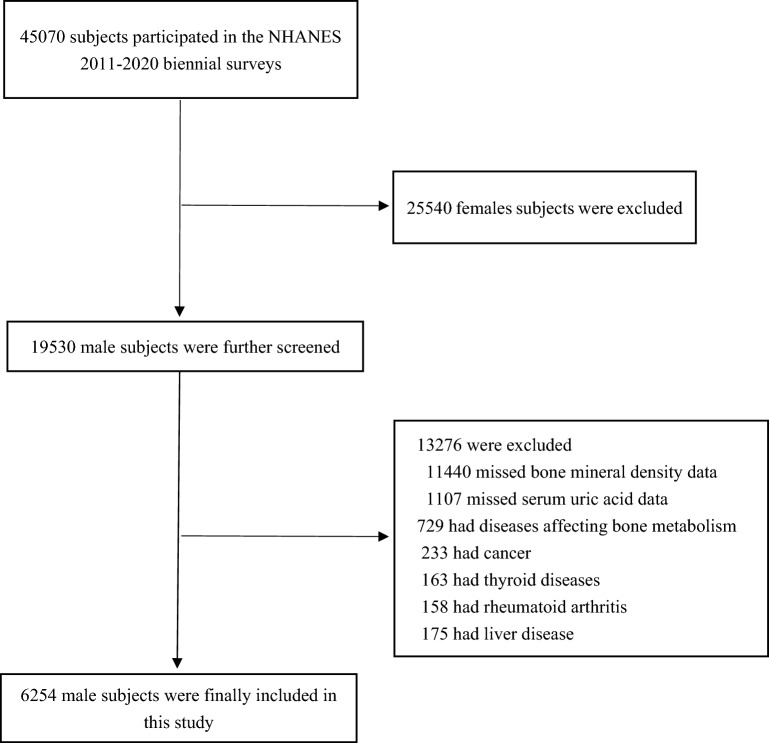
Flowchart of participants selection. BMD, bone mineral density. SUA, serum uric acid.

The NHANES is a representative survey of the national American population through a complex, multistage, and probability sampling design, which provides general health and nutritional status for the civilian and noninstitutional population of the United States. The data from the NHANES 2011–2012, 2013–2014, 2015–2016, and 2017–2020 cycles were combined in this study. The subjects were American men. The participants with missing lumbar spine BMD data (n = 11,440), missing SUA data (n = 1107), and diseases affecting BMD (n = 729) which included cancer patients (n = 233), thyroid disease (n = 163), rheumatoid arthritis (n = 158), and liver disease (n = 175) were excluded. Finally, 6,254 eligible subjects were enrolled in this study. The flow chart of participant selection is presented in Fig. 1.

### Variables

SUA and gout were the exposure variables in this study. SUA was measured as a part of the routine serum biochemistry profile using the Beckman Coulter UniCel^®^ DxC800 with the timed endpoint method. SUA was oxidized by uricase to produce allatoin and hydrogen peroxide. Catalyzed by peroxidase, the hydrogen peroxide reacted with 4-aminoantipyrine (4-AAP) and 3, 5-dichloro-2-hydroxybenzene sulfonate (DCHBS) to produce a colored product. A system was used to monitor the change in absorbance at 520 nm at a fixed time interval, and the change in absorbance was directly proportional to the concentration of uric acid in the sample. Gout was confirmed by trained interviewers using a structured questionnaire collected at home through a computer-assisted personal interview (CAPI) system. The outcome variable was the lumbar spine BMD measured by dual-energy X-ray absorbtiometry (DXA). The measurement site was the lumbar spine, and the scans were all performed with a Hologic QDR 4500A sector beam densitometer (Hologic, Inc., Bedford, Massachusetts). All measured BMD values were collected and standardized by professionals.

In addition, the following covariates were included: age, sex, race/Hispanic origin, body mass index (BMI), poverty-to-income ratio (PIR), physical activity, blood urea nitrogen, total protein, total cholesterol, serum phosphorus, serum calcium, drinking behavior, and smoking behavior. The details on SUA, gout, and lumbar spine BMD measurement procedures and other covariate acquisition procedures could be found on the NHANES website.

### Statistical analysis

For the included data, the software packages R and EmpowerStats were used for analysis, and P values < 0.05 were considered statistically significant. The data were expressed as mean ± standard error (SE) for continuous variables and as percentages for categorical variables. To compare the differences between groups, the weighted chi-square test and the weighted linear regression model were used for continuous variables and classification, respectively. The participants were characterized by quartiles of SUA (Category 1: 0.4–4.9 mg/dL; Category 2: 5.0–5.7 mg/dL; Category 3: 5.8–6.6 mg/dL; and Category 4: > 6.7 mg/dL). A weighted multiple logistic regression model was used to assess the association between SUA and lumbar spine BMD. Multiple regression analyses stratified by age and race were performed. Subsequently, smooth curve fitting and generalized additive models were used to analyze the nonlinear relationship between SUA and lumbar spine BMD. Finally, threshold effect and saturation effect analyses were used to calculate the inflection point of the relationship between SUA and lumbar spine BMD, and a segmented linear regression model was established on both sides of the inflection point.

### Ethics statement

The NCHS Ethics Review Board granted approval for the conduct of NHANES, and written informed consent was obtained from all participants. We confirm that all methods were performed by the relevant guidelines and regulations.

## Results

This study included a total of 6254 male participants who were aged between 12 and 80 years and classified according to the SUA quartiles (Category 1: 0.4–4.9 mg/dL; Category 2: 5.0–5.7 mg/dL; Category 3: 5.8–6.6 mg/dL; and Category 4: > 6.7 mg/dL). The inclusion and exclusion processes were shown in Table 1. Baseline characteristics differed significantly between the SUA quartiles except for total serum calcium, serum phosphorus, and protein. Compared with the other subgroups, participants in the highest quartile of SUA were more likely to be non-Hispanic White and non-Hispanic Black and had higher age, BMI, PIR, blood urea nitrogen, cholesterol, and lumbar spine BMD. In addition, individuals in the highest quartile of SUA might have better education, but they were more likely to drink and smoke.

**Table 1:** Characteristics of the study population based on SUA quartiles.

SUA (serum uric acid)	Total	Q1(0.4–4.9)	Q2(5.0–5.7)	Q3(5.8–6.6)	Q4(> 6.7)	P value
Number of subjects (n)	6254	1404	1612	1670	1568	
Age (years)	34.36 ± 16.25	32.87 ± 17.51	33.61 ± 16.38	34.70 ± 15.94	36.10 ± 15.09	< 0.001
BMI (kg/m^2^)	27.32 ± 6.45	24.34 ± 5.37	26.06 ± 5.65	27.90 ± 5.88	30.67 ± 7.02	< 0.001
PIR	2.44 ± 1.57	2.26 ± 1.50	2.49 ± 1.57	2.44 ± 1.57	2.55 ± 1.60	< 0.001
Moderate recreational activities (minutes)	69.27 ± 46.02	69.79 ± 49.48	68.26 ± 44.39	69.79 ± 45.55	69.30 ± 44.96	0.208
Lumbar spine BMD (g/cm^2^)	1.02 ± 0.17	0.98 ± 0.19	1.01 ± 0.16	1.03 ± 0.16	1.04 ± 0.16	< 0.001
Blood urea nitrogen (mmol/L)	4.66 ± 1.63	4.50 ± 1.55	4.59 ± 1.58	4.62 ± 1.52	4.90 ± 1.82	< 0.001
Serum total calcium (mmol/L)	2.37 ± 0.09	2.37 ± 0.09	2.37 ± 0.08	2.37 ± 0.09	2.37 ± 0.09	0.331
Cholesterol (mmol/L)	4.70 ± 1.08	4.47 ± 1.03	4.59 ± 1.04	4.73 ± 1.09	4.97 ± 1.11	< 0.001
Serum phosphorus (mmol/L)	1.25 ± 0.23	1.30 ± 0.26	1.26 ± 0.23	1.24 ± 0.22	1.22 ± 0.20	< 0.001
Total protein (g/L)	72.37 ± 4.48	71.46 ± 4.39	72.16 ± 4.38	72.42 ± 4.38	73.36 ± 4.55	< 0.001
Race/Hispanic origin (%)						< 0.001
Mexican American	1018 (16.28%)	257 (18.30%)	276 (17.12%)	281 (16.83%)	204 (13.01%)	
Other hispanic	623 (9.96%)	138 (9.83%)	172 (10.67%)	177 (10.60%)	136 (8.67%)	
Non-hispanic white	2057 (32.89%)	410 (29.20%)	540 (33.50%)	572 (34.25%)	535 (34.12%)	
Non-hispanic black	1444 (23.09%)	381 (27.14%)	345 (21.40%)	356 (21.32%)	362 (23.09%)	
Other race—including multi-racial	1112 (17.78%)	218 (15.53%)	279 (17.31%)	284 (17.01%)	331 (21.11%)	
Education level (%)						< 0.001
Less than 9th grade	335 (5.36%)	81 (5.77%)	80 (4.96%)	93 (5.57%)	81 (5.17%)	
9–11th grade	616 (9.85%)	139 (9.90%)	131 (8.13%)	176 (10.54%)	170 (10.84%)	
High school graduate	1099 (17.57%)	215 (15.31%)	284 (17.62%)	288 (17.25%)	312 (19.90%)	
College degree or above	2557 (40.89%)	479 (34.12%)	668 (41.44%)	688 (41.20%)	722 (46.05%)	
Not reported	1647 (26.34%)	490 (34.90%)	449 (27.85%)	425 (25.45%)	283 (18.05%)	
4/5 or more drinks every day (%)						< 0.001
Yes	820 (13.11%)	161 (11.47%)	185 (11.48%)	215 (12.87%)	259 (16.52%)	
No	3393 (54.25%)	646 (46.01%)	900 (55.83%)	944 (56.53%)	903 (57.59%)	
Not reported	2041 (32.64%)	597 (42.52%)	527 (32.69%)	511 (30.60%)	406 (25.89%)	
Smoked at least 100 cigarettes in life (%)						< 0.001
Yes	2198 (35.15%)	487 (34.69%)	531 (32.94%)	570 (34.13%)	610 (38.90%)	
No	2660 (42.53%)	473 (33.69%)	685 (42.49%)	765 (45.81%)	737 (47.00%)	
Not reported	1396 (22.32%)	444 (31.62%)	396 (24.57%)	335 (20.06%)	221 (14.09%)	

Mean ± SD for continuous variables: the P value was calculated by the weighted linear regression model. (%) for categorical variables: the P value was calculated by the weighted chi-square test.

SUA: serum uric acid; PIR: poverty income ratio; BMD: bone mineral density; BMI: body mass index.

**Figure 2:**
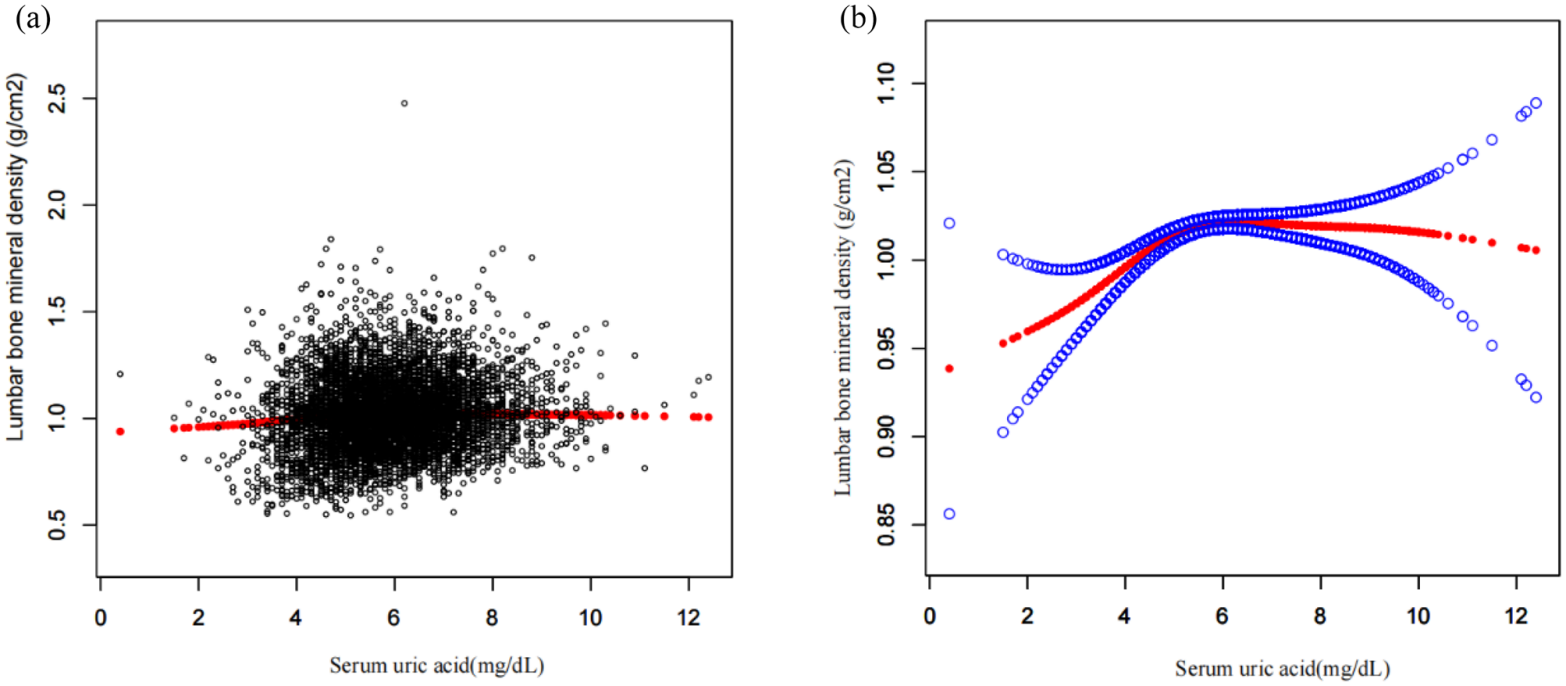
Relationship between serum uric acid and lumbar spine bone mineral density. (**a**) Each black dot represents a sample. (**b**) The solid line indicates a smooth curve fit between the variables. The blue band indicates the 95% confidence interval of the fit. Adjusted for age, Race/Hispanic origin, drinking behavior, smoking behavior, physical activity, BMI, PIR, total protein, serum calcium, cholesterol, serum phosphorus and blood urea nitrogen.

The correlation between SUA and lumbar spine BMD in American men was assessed by multiple regression analysis, and the results were shown in Table 2. In the uncorrected model, SUA was positively associated with lumbar spine BMD in American men (β = 0.014, 95% CI 0.011–0.017, P < 0.001). After correction for covariates, the correlation between SUA and lumbar spine BMD was also significant in Model 2 (β = 0.013 95% CI 0.010–0.017, P < 0.001) and Model 3 (β = 0.006 95% CI 0.003–0.009, P < 0.001). The smooth curve fitting of the relationship between SUA and lumbar spine BMD was shown in Fig. 2. After the subsequent conversion of SUA from a continuous variable to a categorical variable (quartile), individuals in the highest quartile had a higher BMD of 0.020 g/cm^2^ than those in the lowest SUA quartile (β = 0.020 95% CI 0.008–0.032, P = 0.003).

**Table 2:** The association between SUA and Lumbar Spine BMD (g/cm^2^) in men.

	Model 1 β (95% CI) P value	Model 2 β (95% CI) P value	Model 3 β (95% CI) P value
SUA	0.014 (0.011, 0.017) < 0.001	0.013 (0.010, 0.017) < 0.001	0.006 (0.003, 0.009) < 0.001
SUA categories			
Q1 (0.4–4.9 mg/dL)	Reference	Reference	Reference
Q2 (5.0–5.7 mg/dL)	0.029 (0.017, 0.041) < 0.001	0.032 (0.021, 0.043) < 0.001	0.019 (0.007, 0.030) 0.001
Q3 (5.8–6.6 mg/dL)	0.036 (0.024, 0.048) < 0.001	0.037 (0.026, 0.048) < 0.001	0.019 (0.008, 0.030) < 0.001
Q4 (> 6.7 mg/dL)	0.048 (0.036, 0.060) < 0.001	0.047 (0.035, 0.058) < 0.001	0.020 (0.008, 0.032) < 0.001
P for trend	< 0.001	< 0.001	0.003
Subgroup analysis stratified by age			
12–19	0.046 (0.039, 0.052) < 0.001	0.024 (0.018, 0.029) < 0.001	0.024 (0.018, 0.029) < 0.001
20–34	− 0.004 (− 0.010, 0.002) 0.194	− 0.002 (− 0.008, 0.004) 0.469	− 0.002 (− 0.008, 0.004) 0.535
35–49	0.001 (− 0.006, 0.007) 0.828	− 0.001 (− 0.008, 0.005) 0.749	− 0.003 (− 0.010, 0.004) 0.415
≥ 50	0.016 (0.010, 0.023) < 0.001	0.016 (0.009, 0.022) < 0.001	0.010 (0.003, 0.018) 0.004
Subgroup analysis stratified by race/ethnicity			
Mexican American	0.009 (0.002, 0.016) 0.015	0.009 (0.002, 0.016) 0.016	0.005 (− 0.002, 0.012) 0.181
Other hispanic	0.011 (0.001, 0.021) 0.034	0.010 (− 0.000, 0.020) 0.057	0.001 (− 0.010, 0.012) 0.879
Non-hispanic white	0.016 (0.010, 0.021) < 0.001	0.016 (0.010, 0.021) < 0.001	0.009 (0.003, 0.015) 0.003
Non-hispanic black	0.015 (0.008, 0.023) < 0.001	0.011 (0.004, 0.018) 0.003	0.003 (− 0.005, 0.011) 0.412
Other race-including multi-racial	0.008 (0.001, 0.016) 0.037	0.009 (0.001, 0.017) 0.026	0.000 (− 0.008, 0.008) 0.948

Model 1: no covariates were adjusted. Model 2: age and race/ethnicity were adjusted. Model 3: age, race/Hispanic origin, drinking behavior, smoking behavior, physical activity, BMI, PIR, total protein, serum calcium, cholesterol, serum phosphorus, blood urea nitrogen.

SUA: serum uric acid. PIR: poverty income ratio. BMI: body mass index.

**Figure 3:**
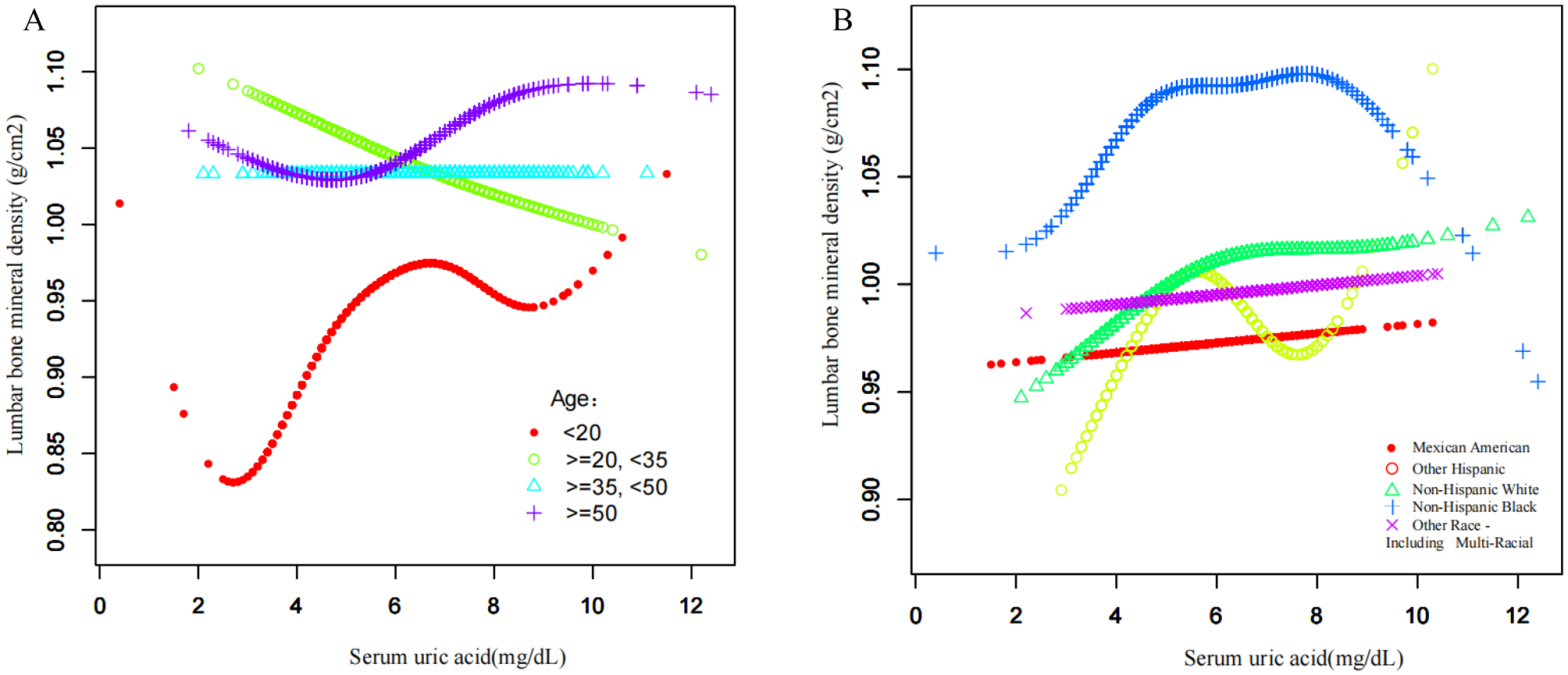
(**A**) The association between serum uric acid and lumbar spine bone mineral density (stratified by age). Adjusted for race/Hispanic origin, drinking behavior, smoking behavior, physical activity, BMI, PIR, total protein, serum calcium, cholesterol, serum phosphorus, blood urea nitrogen. (**B**) The association between serum uric acid and lumbar spine bone mineral density (stratified by race/Hispanic origin). Adjusted for age, drinking behavior, smoking behavior, physical activity, BMI, PIR, total protein, serum calcium, cholesterol, serum phosphorus, blood urea nitrogen.

In the subgroup analysis stratified by age and race, as shown in Table 2, it was found that the positive association between SUA and lumbar spine BMD remained significant in the 12–19 years age group (β = 0.024 95% CI 0.018–0.029, P < 0.001) and in the > 50 years age group (β = 0.010 95% CI 0.003–0.018, P = 0.004) but not in the 20–34 years age group (β = − 0.002 95% CI − 0.008 to 0.004, P = 0.535) and 35–50 years age group (β = − 0.003 95% CI − 0.010 to 0.004, P = 0.415). The positive association between SUA and lumbar spine BMD was more pronounced in non-Hispanic White (β = 0.009 95% CI 0.003–0.015, P = 0.003) but not in Mexican Americans (β = 0.005 95% CI − 0.002 to 0.012, P = 0.181), other Hispanic (β = 0.001 95% CI − 0.010 to 0.012, P = 0.879), non-Hispanic Black (β = 0.003 95% CI − 0.005 to 0.011, P = 0.412), and other races (including the multiracial population) (β = 0.000 95% CI − 0.008 to 0.008, P = 0.948). The smooth curve fitting and the generalized additive model used to characterize the nonlinear relationship between SUA and lumbar spine BMD were shown in Figs. 2 and 3A and B. Data analysis also revealed that in the > 50 years age group, the relationship between SUA and lumbar spine BMD was a positive U-shaped curve with an inflection point of 4.5 mg/ml determined by using a two-stage linear regression model; however, in the 12–19 years age group, the relationship between SUA and lumbar spine BMD was an inverted U-shaped curve with an inflection point of 5.1 mg/ml determined by using a two-stage linear regression model. This inverted U-shaped curve was also present in other Hispanic and non-Hispanic Black. By analyzing the NHANES data for the whole population (including women) from 2011 to 2016 (Table 3), this study found a correlation between the presence or absence of gout in individuals and the lumbar spine BMD, with individuals with gout having a higher BMD of 0.038 g/cm^2^ than those without gout (β = 0.038 95% CI 0.018–0.059, P < 0.001). In the analysis stratified by gender, age, and race, this positive correlation was more pronounced in men, the 20–34 years age group, the > 50 years age group, Mexican Americans, and other Hispanic populations. We also performed a multiple regression analysis of the association between SUA and lumbar spine BMD in adolescent males aged 12-19 years (Supplementary Table 1).

**Table 3:** The association between gout and lumbar spine BMD (g/cm^2^) in men.

	Model 1 β (95% CI) P value	Model 2 β (95% CI) P value	Model 3 β (95% CI) P value
Doctor ever told you that you had gout			
No	Reference	Reference	Reference
Yes	0.031 (0.010, 0.052) 0.004	0.038 (0.017, 0.058) < 0.001	0.038 (0.018, 0.059) < 0.001
P for trend	< 0.001	< 0.001	0.007
Subgroup analysis stratified by gender			
Men			
No gout	Reference	Reference	Reference
Have gout	0.053 (0.027, 0.079) < 0.001	0.053 (0.027, 0.078) < 0.001	0.045 (0.020, 0.071) < 0.001
Women			
No gout	Reference	Reference	Reference
Have gout	− 0.024 (− 0.063, 0.015) 0.229	− 0.010 (− 0.048, 0.029) 0.618	− 0.004 (− 0.043, 0.034) 0.830
Subgroup analysis stratified by age			
20–34			
No gout	Reference	Reference	Reference
Have gout	0.070 (− 0.001, 0.141) 0.053	0.063 (− 0.007, 0.132) 0.078	0.072 (0.003, 0.142) 0.042
35–49			
No gout	Reference	Reference	Reference
Have gout	0.006 (− 0.030, 0.042) 0.745	0.002 (− 0.033, 0.037) 0.917	0.019 (− 0.017, 0.055) 0.304
≥ 50			
No gout	Reference	Reference	Reference
Have gout	0.062 (0.032, 0.093) < 0.001	0.057 (0.027, 0.087) < 0.001	0.034 (0.005, 0.064) 0.023
Subgroup analysis stratified by race/ethnicity			
Mexican American			
No gout	Reference	Reference	Reference
Have gout	0.051 (− 0.019, 0.122) 0.155	0.076 (0.006, 0.147) 0.033	0.071 (0.001, 0.142) 0.049
Other hispanic			
No gout	Reference	Reference	Reference
Have gout	0.079 (− 0.007, 0.166) 0.073	0.094 (0.007, 0.180) 0.034	0.091 (0.005, 0.177) 0.038
Non-hispanic white			
No gout	Reference	Reference	Reference
Have gout	0.018 (− 0.013, 0.050) 0.259	0.027 (− 0.005, 0.059) 0.094	0.030 (− 0.002, 0.063) 0.065
Non-hispanic black			
No gout	Reference	Reference	Reference
Have gout	0.039 (− 0.007, 0.085) 0.010	0.057 (0.010, 0.103) 0.017	0.046 (− 0.001, 0.093) 0.056
Other race—including multi-racial			
No gout	Reference	Reference	Reference
Have gout	0.035 (− 0.027, 0.096) 0.273	0.045 (− 0.016, 0.107) 0.150	0.051 (− 0.010, 0.113) 0.104

Model 1: no covariates were adjusted. Model 2: age and race/ethnicity were adjusted. Model 3: age, race/Hispanic origin, drinking behavior, smoking behavior, physical activity, BMI, PIR, total protein, serum calcium, cholesterol, serum phosphorus, blood urea nitrogen.

SUA: serum uric acid. PIR: poverty income ratio. BMI: body mass index.

## Discussion

In this cross-sectional study, the data from a large multilevel sample of the US population were evaluated to explore the relationship between SUA and BMD in American men, and a positive correlation between them was found. This association was significantly expressed in men in the 12–19 years age group (β = 0.024 95% CI 0.018–0.029, P < 0.001), ≥ 50 years age group (β = 0.010 95% CI 0.003–0.077, P = 0.004), and non-Hispanic White (β = 0.009 95% CI 0.003–0.015, P = 0.003). In addition, a positive association between gout and lumbar spine BMD was also found, with those with gout having a higher BMD of 0.038 g/cm^2^ than those without gout (β = 0.038 95% CI 0.018- 0.059, P < 0.001).

Osteoporosis is a systemic disease characterized by a decrease in both bone mass per unit volume and bone strength, which predisposes affected bones to fractures. It is currently one of the leading causes of morbidity and mortality in the elderly worldwide^13^. Epidemiological investigations and laboratory studies in recent years have indicated that SUA may be involved in a variety of biological processes, associated with diseases such as gout, obesity, and chronic kidney disease, and also a protective factor for BMD^11,12,14^. Previous studies found a positive correlation between SUA and BMD, but most of them revealed that this correlation was mostly present in older men and adolescents, and was not seen in women, especially postmenopausal women^15–18^. A study using the early NHANES data (1996–2006) showed a positive association between SUA and lumbar spine BMD in older adults. The association was in an inverted U-shaped curve in Black^15^. A prospective cohort study of fracture cases from the United States included 1,680 men, and the analysis group contained 387 men who had non-spine fractures (73 hips) and 1,383 randomized samples. The analysis discovered that higher SUA levels were associated with a reduced risk of non-spine fractures, i.e., uric acid had a protective effect on bone density^19^. However, an analysis of data from the NHANES (1996–2006) by Li et al. found no association between SUA and BMD in American men (β = − 0.003 95% CI − 0.007 to 0.002), even after correction for relevant confounding factors^20^. In addition, a cross-sectional study from China also reported a positive association between SUA and BMD in postmenopausal women (n = 4256) but not in men (n = 943)^21^. They found no correlation between SUA and BMD in men, which is different from the results of this study.

Several aspects can explain this difference. First, their studies were comparative studies from different SUA cohorts and might not have been subjected to standardized regression analysis and adjustment for confounding factors. Second, their studies performed subgroup analyses by different grouping methods, which may also account for the difference. For example, Li et al.^20^ divided the population into three groups by age i.e., < 40 years age group, 40–59 years age group, and ≥ 60 years age group, and they also divided the population into < 7 mg/dL and ≥ 7 mg/dL groups based on SUA levels. Third, their study had a limited number of participants, which may lead to unstable results. In conclusion, heterogeneity among these studies, such as differences in methodological design, included populations, stratified analysis methods, and controlled confounding variables, may explain the existence of controversy.

This cross-sectional study demonstrated a positive association between SUA and BMD in American men. Several possible mechanisms were proposed to explain this association. First, individuals with high uric acid may live in better conditions and consume more nutrients than those with low uric acid^11,14^. Previous studies have considered SUA as a nutritional marker that can represent the nutritional status of an individual. The main sources of uric acid are purine-rich foods such as meat, seafood, and purine-rich vegetables, all of which are also high in protein. In puberty when bone mass is needed for growth and development and in old age when bone mass is lost, people with better living conditions and better nutritional intake may have higher BMD than those with low uric acid^22^. A study from the United States has also suggested that nutritional supplements are key to preventing osteoporosis^10^. Second, the protective effect of SUA on BMD may be related to its antioxidant capacity. SUA is a powerful endogenous antioxidant that clears peroxyl alone (RO2.), hydroxyl alone (.OH), and singlet oxygen radicals, respectively^10,23^. Low SUA concentrations reduce the ability to resist oxidative stress, thereby promoting osteoclast differentiation and reducing osteoblast activity, resulting in enhanced bone resorption and bone loss^24^. Another study has shown that uric acid plays an important role in the expansion of human bone marrow mesenchymal stem cells and promotes osteogenic differentiation^25^. Third, lower SUA concentrations were associated with lower serum parathyroid hormone (PTH) levels and higher serum TRACP5b levels. This would counter support and explain the idea that lower SUA concentrations enhance bone resorption, as reflected by an increase in serum TRACP5b levels, and this leads to greater loss of BMD and elevated serum calcium levels, causing a decrease in serum PTH levels as a result of negative feedback^26^. Previous studies have also found that PTH, as a metabolic factor, may affect the clearance of SUA, leading to an increase in SUA levels, and is involved in the relationship between SUA and bone metabolism^27,28^. In addition, people with high SUA levels have more lean body mass without many changes in fat mass. Some studies suggest that there is a positive correlation between lean body mass and BMD^29^. Despite these speculations and findings, the exact mechanism underlying the positive association between SUA and BMD remains uncertain and requires further investigation.

It could be known from the results of smooth curve fitting (Fig. 2) and threshold effect analysis (Table 4) that when SUA levels were < 5 mg/dL, BMD increased with SUA and there was a positive correlation between SUA and BMD; but when SUA levels were ≥ 5 mg/dL, the positive correlation between SUA and BMD was no longer significant. Considering the influence of SUA on other diseases, this saturation effect suggests that raising the level of SUA moderately within its normal range may be beneficial to bone health and will not have adverse effects on other systems. Then, a stratified analysis based on race was conducted in this study, finding that the positive correlation between SUA and lumbar spine BMD was significant in non-Hispanic White but not in other races. The smooth curve fitting (Fig. 3) indicated that the relationship between SUA and BMD in non-Hispanic Black was an inverted U-shaped curve, which is the same as a previous study. Genetic factors, lifestyle differences, and other factors may provide possible explanations for ethnic differences in this relationship^15^. Figure 3A suggests that serum uric acid is negatively correlated with lumbar spine BMD in the 20–35 age group, where BMD is stable, whereas this relationship becomes reversed and unstable with saturation in the adolescent and elderly populations. Figure 3B suggests that in non-Hispanic blacks, serum uric acid and lumbar spine BMD show a clear inverted U-shaped curve, which correlates with the fact that blacks are more physically active and require more nutrition.

**Table 4:** Analysis of threshold and saturation effects between SUA and lumbar spine BMD.

Outcome	Lumbar spine BMD
Model 1	
A straight-line effect	0.006 (0.003, 0.009) 0.0006
Model 2	
Fold point (K)	5
< K-segment effect 1	0.027 (0.015, 0.039) < 0.0001
> K-segment effect 2	0.001 (− 0.003, 0.006) 0.5198
Effect difference between two segments	− 0.026 (− 0.040, − 0.012) 0.0002
The predicted value of the equation at the breakpoint	1.013 (1.007, 1.020)
Log-likelihood ratio test	< 0.001

A large number of research samples are needed to explain the special relationship between SUA and BMD in the Black population. A stratified analysis based on age was also conducted, concluding that the positive correlation between SUA and BMD often occurred in people aged 12–19 years and ≥ 50 years. Teenagers need to grow and develop, while elderly individuals will experience rapid bone loss. Hence, both groups are in a period of extreme need for bone mass. Many studies have found that there is a positive correlation between SUA and BMD in male adolescents or elderly men, which is consistent with this research^15,16^. In the smooth curve fitting, it was found that the relationship between SUA and BMD in the male population > 50 years old was in a positive U-shape. The impact of age on this relationship implies that it is particularly important to adjust the level of SUA in youth and old age. To the best of our knowledge, this is the first study using the NHANES data (2010–2020) to obtain a positive correlation between SUA and BMD in American men.

This study has several advantages. First, it was based on a large sample of data from the NHANES. The sample in this study was a multilayer random one with highly reliable and standardized data that could be representative of the general population in the United States. Second, because cancer, liver disease, thyroid disease, and rheumatoid arthritis affect bone metabolism, patients with these diseases were excluded during the inclusion of the population. Also, many important confounding factors, including age, race, drinking behavior, smoking behavior, BMI, serum urea nitrogen, and total protein, were excluded or controlled. Furthermore, the findings of this study are more reliable because the population in this study was not affected by menopause, estrogen, or pregnancy. Finally, to the extent of our knowledge, the present study is the first to analyze the NHANES (2011–2020) data to investigate the relationship between SUA and BMD in American men, with multiple regression analyses stratified by age and race.

Of course, there are also some shortcomings in this study. First, because it is a cross-sectional study, no causal relationship can be inferred, and more longitudinal studies are needed to verify this relationship. Second, this study only investigated the relationship between SUA and lumbar spine BMD and did not examine the relationship between SUA and femoral BMD due to the lack of data, resulting in an incomplete result. It has been shown that the relationship between SUA and BMD is different in two different skeletal sites, i.e., the femur and the lumbar spine, owing to the influence of mechanical factors^30^. Hence, more studies are needed in the future to explore the relationship between SUA and BMD at different skeletal sites in men. Finally, other potential confounders that were not adjusted in this study could still lead to bias.

## Conclusions

This study showed a positive correlation between SUA and lumbar spine BMD in American men, but the correlation was no longer significant when SUA was ≥ 5 mg/dL. Confounding factors such as race and age may influence the positive correlation, and after the analysis stratified by age and race, the positive correlation between SUA and lumbar spine BMD was found to remain significant in the 12–19 years age group, the > 50 years age group, and non-Hispanic White. This gives clinicians some insight into how to monitor SUA levels to predict BMD levels during adolescence when bone is urgently needed for growth and development and during old age when bone loss is rapid.

### Supplementary Information

Supplementary Table 1.

## Data Availability

The datasets generated and analyzed during the current study can be available from the corresponding author on reasonable request. The datasets for this study can be found at http://www.cdc.gov/nchs/nhanes/.
