# Supplementary material for: Association between serum uric acid and bone mineral density in males from NHANES 2011–2020
Source: Sci Rep. 2024 Feb 21;14:4292. doi: 10.1038/s41598-024-52147-8 (PMC10881460; doi:10.1038/s41598-024-52147-8)
Supplement: Supplementary file 1 — Supplementary Table 1. [file 41598_2024_52147_MOESM1_ESM.docx]

Supplementary tables 1 The association between SUA and Lumbar Spine BMD (g/cm2) in men aged 12-19 years

|  | Model 1 β (95% CI) P value | Model 2 β (95% CI) P value | Model 3 β (95% CI) P value |
| --- | --- | --- | --- |
| SUA | 0.046 (0.039, 0.052) <0.001 | 0.020 (0.014, 0.025) <0.001 | 0.010 (0.004, 0.016) 0.001 |
| SUA categories | | | |
| Q1(0.4-4.7) | Reference | Reference | Reference |
| Q2(4.8-5.4) | 0.090 (0.067, 0.112) <0.001 | 0.044 (0.025, 0.063) <0.001 | 0.036 (0.018, 0.055) <0.001 |
| Q3(5.5-6.2) | 0.111 (0.090, 0.133) <0.001 | 0.055 (0.036, 0.073) <0.001 | 0.041 (0.022, 0.059) <0.001 |
| Q4(6.3-11.5) | 0.161 (0.139, 0.182) <0.001 | 0.081 (0.063, 0.100) <0.001 | 0.050 (0.031, 0.070) <0.001 |
| P for trend | <0.001 | <0.001 | <0.001 |
| Subgroup analysis stratified by age | | | |
| 12-15 | 0.048 (0.040, 0.055) <0.001 | 0.032 (0.025, 0.039) <0.001 | 0.020 (0.013, 0.028) <0.001 |
| 16-19 | 0.004 (-0.005, 0.012) 0.379 | 0.004 (-0.004, 0.013) 0.313 | -0.005 (-0.015, 0.004) 0.274 |
| Subgroup analysis stratified by race/ethnicity | | | |
| Mexican American | 0.024 (0.010, 0.038) <0.001 | 0.006 (-0.006, 0.017) 0.347 | 0.002 (-0.010, 0.014) 0.719 |
| Other Hispanic | 0.063 (0.041, 0.085) <0.001 | 0.042 (0.022, 0.062) <0.001 | 0.021 (-0.001, 0.043) 0.058 |
| Non-Hispanic White | 0.056 (0.044, 0.067) <0.001 | 0.042 (0.022, 0.062) <0.001 | 0.025 (0.013, 0.036) 0.00004 |
| Non-Hispanic Black | 0.043 (0.029, 0.056) <0.001 | 0.021 (0.010, 0.032) <0.001 | 0.010 (-0.004, 0.023) 0.153 |
| Other Race - Including Multi-Racial | 0.029 (0.010, 0.048) 0.003 | -0.005 (-0.022, 0.012) 0.588 | -0.020 (-0.037, -0.002) 0.026 |

Model 1: no covariates were adjusted. Model 2: age and race/ethnicity were adjusted. Model 3: age, Race/Hispanic origin, physical activity , BMI, PIR, total protein, serum calcium, cholesterol, serum phosphorus, blood urea nitrogen. Abbreviation: SUA: serum uric acid. PIR poverty income ratio.

BMI: body mass index.

We performed a multiple regression analysis of the association between SUA and lumbar spine BMD in adolescent males aged 12-19 years. We found a positive association between sUA and lumbar spine BMD in the uncorrected model (β= 0.046, 95% confidence interval: 0.039-0.052, P<0.001). After correcting for confounders, this positive correlation remained in model 2 (β= 0.020, 95% CI:0.014-0.025, P<0.001) and model 3 (β= 0.010, 95% confidence interval: 0.004-0.016, P=0.001). After converting SUA from a continuous variable to a categorical variable (quartiles), individuals in the highest quartile had 0.050 g/cm² higher BMD than individuals in the lowest SUA quartile.

In the subgroup analyses stratified by age and race/ethnicity reported in Supplementary Table 1, the positive association between SUA and lumbar spine BMD remained significant in adolescents aged 12-15 years, Non-Hispanic White, but not in adolescents aged 16-19 years, Mexican American, Other Hispanic, Non- Hispanic Black, and a negative association in Other Race - Including Multi-Racial (β=-0.020, 95% CI:-0.037-0.002, P=0.026).
